# Comparison of the efficacy and safety between oral sulfate tablet and polyethylene glycol for bowel preparation before colonoscopy according to age

**DOI:** 10.1097/MD.0000000000029884

**Published:** 2022-07-08

**Authors:** Jae Hyun Kim, Yong Eun Park, Tae Oh Kim, Jongha Park, Gyu Man Oh, Won Moon, Seun Ja Park

**Affiliations:** a Department of Internal Medicine, Kosin University College of Medicine, Busan, Korea; b Department of Internal Medicine, Inje University Haeundae Paik Hospital, Inje University College of Medicine, Busan, Korea.

**Keywords:** bowel preparation, colonoscopy, elderly, oral sulfate tablet, polyethylene glycol

## Abstract

**Background:**

Recently, a novel oral sulfate tablet (OST) has been introduced for bowel preparation before colonoscopy. However, whether elderly patients can take OST is not yet clear, as OST consists of 28 tablets. We aimed to compare the efficacy and safety of OST and polyethylene glycol (PEG) for bowel preparation for colonoscopy according to age.

**Methods:**

We randomly divided subjects into an OST group and a PEG group and compared Boston Bowel Preparation Score (BBPS), bubble score, patient compliance and satisfaction, and safety between the 2 groups according to age (under 65 years of age vs 65 years of age and older).

**Results:**

Among the 179 participants, 61 were 65 years of age and older. The BBPS and bubble score of the OST group were better than that of the PEG group, regardless of age. The satisfaction of the OST group was better than that of the PEG group, regardless of age. The compliance was not different between the 2 groups; however, the OST group under 65 years of age had a higher rate of completing the dose within 2 hours compared with the PEG group under 65 years of age. Adverse events including abdominal distension, abdominal pain, nausea, or vomiting were not different between the 2 groups.

**Conclusion:**

The use of OST for bowel preparation before colonoscopy is as effective and safe as PEG, and these results were consistent in elderly people 65 years of age and older.

## 1. Introduction

Colonoscopy is a useful tool to detect and remove colorectal polyps, a precursor lesion of colorectal cancer.^[1]^ High-quality bowel preparation is essential for complete examination of the colon mucosa during colonoscopy.^[2,3]^ However, bowel preparation is inadequate for up to one-third of colonoscopic examinations.^[4,5]^ Inadequate bowel preparation can decrease the adenoma detection rates and increase both the risk of complications following the colonoscopy as well as the procedure time.^[6]^ A number of laxatives have been developed to improve the quality of bowel preparation, and a recent guideline provided data on the efficacy and safety of validated laxatives, including high volume polyethylene glycol (PEG), low-volume PEG plus adjuvants, magnesium citrate plus picosulfate, and oral sulfate solution.^[7]^ However, the taking these laxatives is not easy, as patients are required to consume an unpleasant tasting solution.^[8–10]^ Several years ago, a tablet preparation of sodium phosphate had been expected to improve the tolerance of patients^[11,12]^; however, it had limitations, with concerns regarding renal safety.^[13]^

Recently, a novel oral sulfate tablet (OST) has been introduced for bowel preparation before colonoscopy. OST is expected to improve the compliance and satisfaction of patients because this treatment, involving tablets plus pure water, replaces the distasteful liquid. OST consists of sodium sulfate, potassium sulfate, and magnesium sulfate, and it also contains simethicone, which helps remove intraluminal air bubbles and therefore improves visualization during the colonoscopy.^[14,15]^ A recent trial reported a similar efficacy and better safety and tolerability of OST compared with oral sulfate solution.^[16]^ However, whether elderly patients can take OST is not yet clear, as OST consists of 28 tablets.

In this study, we aimed to compare the efficacy, safety, and patient compliance and satisfaction of OST to the commonly used laxative PEG for bowel preparation before colonoscopy according to age.

## 2. Methods

### 2.1. Study design

This study was a prospective, multicenter, randomized controlled trial conducted at the Kosin University Gospel Hospital and the Inje University Haeundae Paik Hospital in Korea between August 2020 and February 2021. The study protocol was approved by the institutional review board of both participating hospitals. This trial is registered with the International Clinical Trials Registry Platform (number KCT0005451).

### 2.2. Patients

Patients >19 years of age undergoing colonoscopy for screening or postpolypectomy surveillance at the endoscopy centers of Kosin University Gospel Hospital and Inje University Haeundae Paik Hospital in Korea were enrolled. Patients with a history of chronic kidney disease, heart failure, ascites, ileus or intestinal obstruction, gastrointestinal bleeding, and allergy to bowel preparation regimen and those who were pregnant were excluded. Patients who had taken laxatives, metoclopramide, tegaserod, or erythromycin within 1 month prior to colonoscopy were also excluded. A study investigator of each hospital explained the aim and contents of the study in detail, and all participants provided written informed consent before enrollment. Clinical information was collected using a questionnaire, including age, sex, height, weight, body mass index, previous colonoscopy experience, and the reason for a colonoscopy. All patients were provided education with a brochure for bowel preparation and colonoscopy, which included the diet schedule (low-fiber diet for 3 days and soft diet for dinner the day before the colonoscopy), methods for taking OST or PEG, the colonoscopy process, and potential adverse events during bowel preparation or colonoscopy. Patients were randomized to either the OST group (Orafang, Pharmbio Korea Co. Ltd, Seoul, Korea) or the PEG group (Coolprep, TaeJoon Pharmaceuticals, Seoul, Korea) using the table of random numbers. Orafang is 28 tablets which consist of sodium sulfate, potassium sulfate, magnesium sulfate, and simethicone, and Coolprep consists of 2 L PEG and ascorbic acid. Patients in the OST group took 14 tablets with water and then drank 1 L of water for an hour between 7 and 10 pm on the day before the colonoscopy, and the remaining 14 tablets in the same way in the morning at least 2 hours before the colonoscopy. Patients in the PEG group ingested 1 L polyethylene glycol solution with ascorbic acid between 7 and 10 pm on the day before the colonoscopy, and the remaining 1 L in the morning at least 2 hours before the colonoscopy.

### 2.3. Data collection

On the day of colonoscopy, all patients were interviewed and filled out a questionnaire, which consisted of questions on compliance, satisfaction, difficulty, taste, willing to switch to another preparation on next examination, total water intake, and adverse events during bowel preparation. Compliance was checked as 2 factors, one based on the total dose that was taken (<25%, 25%–50%, 50%–75%, 75%–99%, or 100%) and another based on a total taking time (per a split dose; <30 minutes, 30 minutes to 1 hour, 1–2 hours, 2–3 hours, or >3 hours) for bowel preparation. Satisfaction was assessed by visual analog scale (0: very bad, 10: excellent). Difficulty of eating was evaluated by a 4-point rating scale (1: ease, 2: relative ease, 3: a little difficult, 4: quite difficult). Taste was also evaluated by a 4-point rating scale (1: good, 2: no taste, 3: not good, but edible, 4: bad). Total water intake was based on the amount of total water intake (<500 mL, 500–1000 mL, >1000 mL). Adverse events during bowel preparation, including abdominal distension, abdominal pain, nausea, and vomiting, were assessed as none, mild, moderate, and severe.

Colonoscopic examinations were performed by 2 experienced endoscopists who were blinded to the group information. During the examination, bowel preparation scale, bubble score, and polyp detection were recorded. The bowel preparation scale was assessed according to the Boston Bowel Preparation Scale (BBPS),^[17]^ and the bubble score was checked using a numeric rating scale (3: excellent, 2: good, 1: poor, 0: inadequate).^[18]^ After examination, an endoscopist evaluated the own satisfaction during colonoscopy using visual analog scale.

### 2.4. Endpoints

The primary endpoint in this study was the comparison of efficacy and safety between the OST and PEG groups according to age. Secondary endpoints included the comparison of compliance, satisfaction, difficulty of eating, taste, willingness to switch to another preparation, polyp detection rate, and adenoma detection rate between the OST and PEG groups according to age.

### 2.5. Statistical analysis

The number of patients in each group was calculated based on expected successful cleansing rates of 85% for both groups, a noninferiority margin of 15% with a power of 0.8, and 1-sided significance level of .025. Considering a dropout rate of 10%, at least 98 subjects in each group were needed for the study. Continuous data with normal distributions were expressed as mean ± standard deviation, and categorical data were presented as the number of subjects (%). Student *t* test and the chi-square test were performed for continuous and categorical variables, as appropriate. *P* values <.05 were considered statistically significant. Statistical analyses were performed using SPSS version 23.0 (IBM Co., Armonk, NY).

## 3. Results

### 3.1. Baseline characteristics

**Figure 1.**
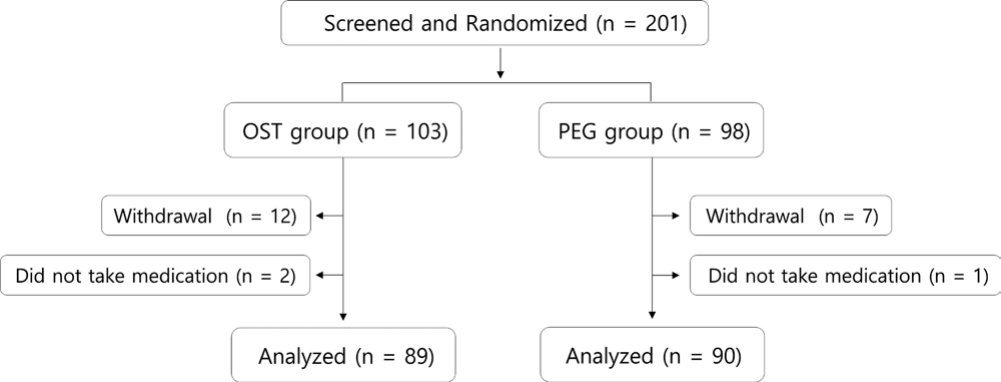
Study flowchart. BBPS = Boston Bowel Preparation Scale, OST = oral sulfate tablet, PEG = polyethylene glycol.

Between August 2020 and February 2021, a total of 201 patients were included in the study and were randomly divided into 2 groups: the OST group and the PEG group. Among the initial patient group, 19 patients withdrew participation for personal reasons and 3 patients did not take medication. Finally, 89 patients in the OST group and 90 patients in the PEG group were included in this study. The study flowchart is presented in Figure 1. The mean patient age was 58.3 ± 10.9 years, and 79 (44.1%) were male. Among the 89 patients, 61 patients (34.1%) were 65 years of age and older. Baseline characteristics are summarized in Table 1.

**Table 1:** Baseline characteristics of participants.

	**OST group** **(N = 89**)	**PEG group** **(N = 90**)
Age (yr)	57.8 ± 10.3	58.8 ± 11.5
<65	60 (67.4)	61 (64.4)
≥65	29 (32.6)	32 (35.6)
Sex		
Male	45 (50.6)	34 (37.8)
Female	44 (49.4)	56 (62.2)
Height	164.9 ± 8.5	163.1 ± 8.8
Weight	65.3 ± 11.3	62.2 ± 11.2
BMI (kg/m^2^)	24.0 ± 2.9	23.3 ± 3.0
Reason for colonoscopy		
Screening	59 (67.0)	63 (70.0)
Postpolypectomy surveillance	29 (33.0)	27 (30.0)
Previous experience of colonoscopy		
No	13 (14.8)	18 (20.0)
Yes	75 (85.2)	72 (80.0)

Values are presented as mean ± standard deviation or n (%).

BMI = body mass index, OST = oral sulfate tablet, PEG = polyethylene glycol.

### 3.2. Compliance and satisfaction of bowel preparation

We divided patients into subgroups based on age under 65 years and 65 years of age and older to evaluate the difference between OST and PEG groups according to age. Information including compliance, satisfaction, difficulty of eating, taste, willingness to switch to another preparation on next examination, and total water intake was collected in a questionnaire submitted by patients on the day of colonoscopy and the data were assessed. As shown in Table 2, the compliance (total taken dose) of patients was not different between the OST and PEG groups, regardless of age. The OST group under 65 years of age had a higher rate of completing the dose within 2 hours compared with the PEG group under 65 years of age (91.6% vs 75.9%, *P* < .001). However, these results were not different between the 2 groups 65 years of age and older (86.2% vs 81.3%, *P* = .979). The satisfaction, level of difficulty, and taste reported by the OST group all scored better than those of the PEG group, regardless of age. Total amounts of water intake were not different between the 2 groups. On the day of colonoscopy, 5 patients (8.5%) under 65 years of age and 6 patients (20.7%) 65 years of age and older in the OST group wanted to switch to another preparation on the next colonoscopy.

**Table 2:** Compliance and satisfaction with bowel preparation according to age.

	Under 65 years of age (N = 118)			65 years of age and older (N = 61)		
	OST group (n = 60)	PEG group (n = 58)	*P* value	OST group (n = 29)	PEG group (n = 32)	*P* value
Compliance (total taking dose)			.365			.614
50%–75%	3 (5.0)	1 (1.7)		0 (0.0)	0 (0.0)	
75%–99%	1 (1.7)	0 (0)		1 (3.4)	3 (9.4)	
100%	56 (93.3)	57 (98.3)		28 (96.6)	29 (90.6)	
Compliance (total taking time)	Based on 14 tablets	Based on 1 L	<.001	Based on 14 tablets	Based on 1 L	.979
<30 min	20 (33.3)	3 (5.2)		4 (13.8)	3 (9.4)	
30 min–1 h	26 (43.3)	33 (56.9)		16 (55.2)	13 (40.6)	
1–2 h	9 (15.0)	8 (13.8)		5 (17.2)	10 (31.3)	
2–3 h	4 (6.7)	13 (22.4)		4 (13.8)	5 (15.6)	
3 h	1 (1.7)	1 (1.7)		0 (0.0)	1 (3.1)	
Satisfaction^*^	8.1 ± 1.6	6.8 ± 2.5	.001	8.4 ± 2.6	6.4 ± 2.4	.003
Difficulty of eating			.002			.003
Ease	23 (38.3)	13 (22.8)		15 (51.7)	4 (12.5)	
Relative ease	24 (40.0)	14 (24.6)		9 (31.0)	11 (34.4)	
A little difficult	12 (20.0)	20 (35.1)		2 (6.9)	11 (34.4)	
Quite difficult	1 (1.7)	10 (17.5)		3 (10.3)	6 (18.8)	
Taste			<.001			<.001
Good	3 (5.0)	6 (10.3)		1 (3.4)	2 (6.3)	
No taste	48 (80.0)	0 (0.0)		20 (69.0)	5 (15.6)	
Not good, but edible	9 (15.0)	45 (77.6)		8 (27.6)	21 (65.6)	
Bad	0 (0.0)	7 (12.1)		0 (0.0)	4 (12.5)	
Total amounts of water intake			.648			.072
<500 mL	4 (6.6)	2 (3.6)		0 (0.0)	0 (0.0)	
500–1000 mL	55 (91.7)	53 (96.4)		22 (75.9)	30 (93.8)	
>1000 mL	1 (1.7)	0 (0.0)		7 (24.1)	2 (6.2)	
Want to switch to another preparation on next colonoscopy	5 (8.5)	31 (54.4)	<.001	6 (20.7)	17 (53.1)	.016

Values are presented as mean ± standard deviation or n (%).

OST = oral sulfate tablet, PEG = polyethylene glycol.

* Visual analog scale score (0–10 points, 0: very bad, 10: excellent).

### 3.3. Adverse events during bowel preparation

We investigated the adverse events experienced by patients during bowel preparation, including abdominal distension, abdominal pain, nausea, and vomiting, which were classified into 4 categories (none, mild, moderate, and severe). The adverse events during bowel preparation between the OST and PEG group were not significantly different, regardless of age (Table 3). Moderate and severe abdominal distension occurred in 11.7% of the OST group under 65 years of age, whereas these events were not reported in the PEG group. In the OST group 65 years of age and older, there were no moderate or severe adverse events.

**Table 3:** Adverse effects during bowel preparation according to age.

**Adverse effects**	**Under 65 years of age (N = 118**)			**65 years of age and older (N = 61**)		
	**OST group** **(n = 60**)	**PEG group** **(n = 58**)	***P* value**	**OST group** **(n = 29**)	**PEG group** **(n = 32**)	***P* value**
Adverse events during bowel preparation						
Abdominal distension			.125			.219
None	34 (56.7)	39 (67.2)		25 (86.2)	23 (71.9)	
Mild	19 (31.7)	19 (32.8)		4 (13.8)	7 (21.9)	
Moderate	6 (10.0)	0 (0.0)		0 (0.0)	1 (3.1)	
Severe	1 (1.7)	0 (0.0)		0 (0.0)	1 (3.1)	
Abdominal pain			.943			.114
None	52 (86.6)	50 (86.2)		29 (100.0)	28 (87.5)	
Mild	7 (11.7)	7 (12.1)		0 (0.0)	4 (12.5)	
Moderate	1 (1.7)	0 (0.0)		0 (0.0)	0 (0.0)	
Severe	0 (0.0)	1 (1.7)		0 (0.0)	0 (0.0)	
Nausea			.450			.938
None	28 (46.7)	33 (56.9)		21 (72.4)	23 (71.9)	
Mild	28 (46.7)	19 (32.8)		8 (27.6)	7 (21.9)	
Moderate	2 (3.3)	4 (6.9)		0 (0.0)	1 (3.1)	
Severe	2 (3.3)	2 (3.4)		0 (0.0)	1 (3.1)	
Vomiting			.755			.613
None	53 (88.3)	49 (84.5)		28 (96.6)	30 (93.8)	
Mild	4 (6.7)	7 (12.1)		1 (3.4)	1 (3.1)	
Moderate	1 (1.7)	1 (1.7)		0 (0.0)	0 (0.0)	
Severe	2 (3.3)	1 (1.7)		0 (0.0)	1 (3.1)	

Values are presented as n (%).

OST = oral sulfate tablet, PEG = polyethylene glycol.

### 3.4. Efficacy and colonoscopy outcomes

**Figure 2.**
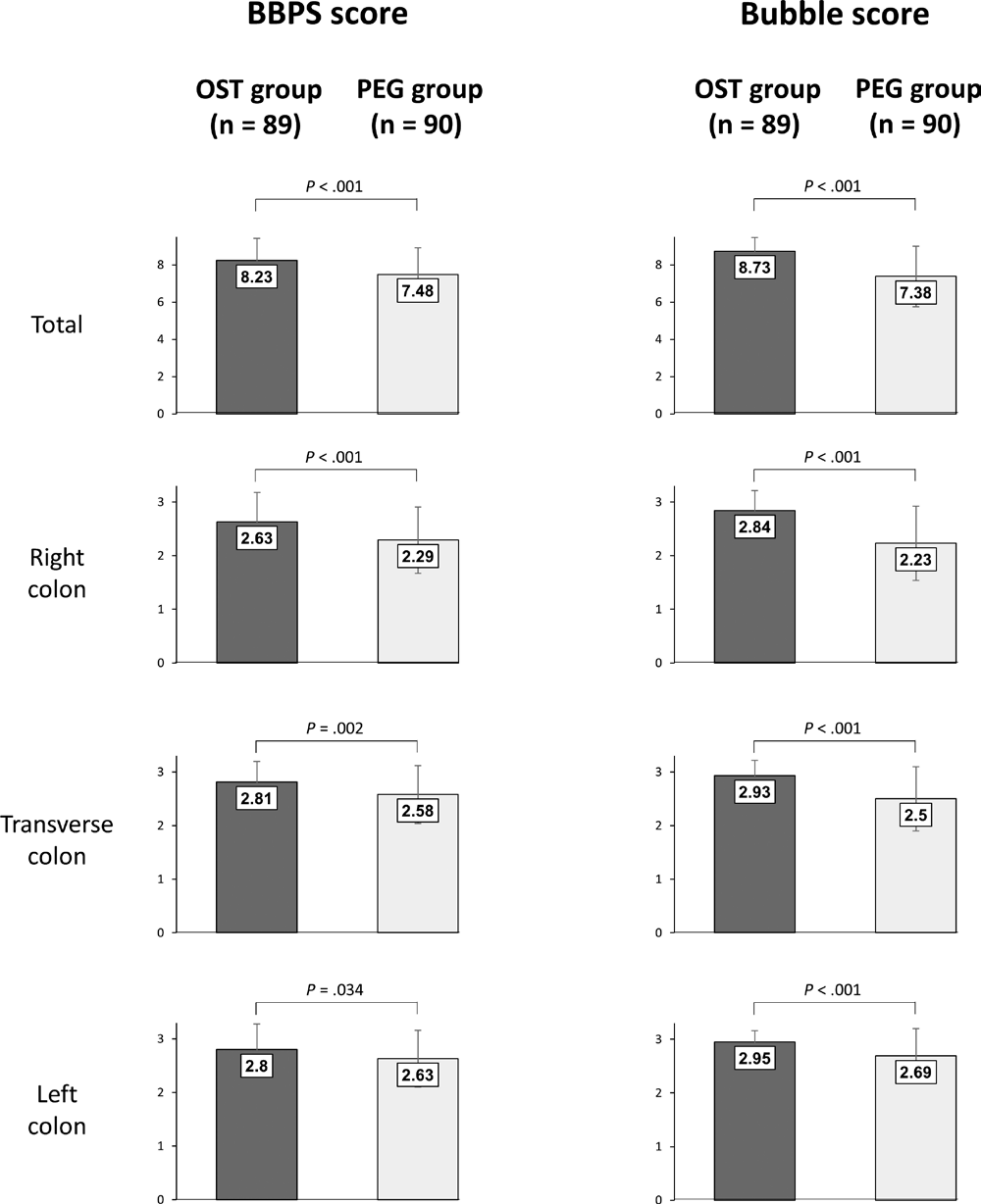
Comparison of the efficacy between OST and PEG groups. BBPS = Boston Bowel Preparation Scale, OST = oral sulfate tablet, PEG = polyethylene glycol.

As shown in Figure 2, the total BBPS score of OST group was better than that of the PEG group (8.23 vs 7.48, *P* < .001) and these results were identical when analyzing the right colon, transverse colon, and left colon. The total bubble score of the OST group was better than that of the PEG group (8.73 vs 7.38, *P* < .001) and the bubble scores of the OST group in right colon, transverse colon, and left colon were all better than those of the PEG group. In the subgroup analysis according to age (Table 4), the BBPS score of the OST group under 65 years of age was better than that of the PEG group under 65 years of age; however, the difference in patients 65 years of age and older was not significant between the 2 groups. The bubble scores of the OST group in both subgroups (under 65 years of age and 65 years of age and older) were better than those of the PEG group. The polyp detection and adenoma detection rate between the 2 groups were not different, regardless of age. Endoscopist satisfaction during colonoscopy was better in the OST group than in the PEG group, although the difference was not significant with patients 65 years of age and older.

**Table 4:** Efficacy and colonoscopy outcomes according to age.

	**Under 65 years of age (N = 118**)			**65 years of age and older (N = 61**)		
	**OST group** **(n = 60**)	**PEG group** **(n = 58**)	***P* value**	**OST group** **(n = 29**)	**PEG group** **(n = 32**)	***P* value**
BBPS score						
Total	8.32 ± 0.97	7.55 ± 1.51	.002	8.07 ± 1.56	7.38 ± 1.29	.062
Right colon	2.65 ± 0.52	2.31 ± 0.65	.002	2.59 ± 0.63	2.25 ± 0.57	.032
Transverse colon	2.82 ± 0.39	2.62 ± 0.56	.029	2.79 ± 0.41	2.50 ± 0.51	.016
Left colon	2.85 ± 0.36	2.66 ± 0.52	.019	2.69 ± 0.66	2.59 ± 0.56	.542
Bubble score						
Total	8.80 ± 0.61	7.47 ± 1.45	<.001	8.59 ± 0.91	7.22 ± 1.93	.001
Right colon	2.87 ± 0.34	2.24 ± 0.63	<.001	2.79 ± 0.41	2.22 ± 0.79	.001
Transverse colon	2.97 ± 0.18	2.52 ± 0.60	<.001	2.86 ± 0.44	2.47 ± 0.62	.006
Left colon	2.97 ± 0.18	2.71 ± 0.50	<.001	2.93 ± 0.26	2.66 ± 0.55	.014
Polyp detection						
Total	34 (56.7)	33 (56.9)	.980	22 (75.9)	19 (59.4)	.187
Right colon	21 (35.0)	18 (31.0)	.698	17 (58.6)	13 (40.6)	.204
Transverse colon	14 (23.3)	10 (17.2)	.495	8 (27.6)	7 (21.9)	.767
Left colon	24 (40.0)	24 (41.4)	.879	13 (47.6)	13 (41.4)	.799
Adenoma detection						
Total	23 (38.3)	23 (39.7)	.883	17 (58.6)	16 (50.0)	.609
Right colon	12 (20.0)	17 (29.3)	.288	12 (41.4)	9 (28.1)	.296
Transverse colon	13 (21.7)	7 (12.1)	.221	6 (20.7)	5 (15.6)	.743
Left colon	8 (13.3)	13 (22.4)	.234	8 (27.6)	7 (21.9)	.767
Endoscopist satisfaction^*^	8.98 ± 0.89	7.72 ± 1.42	<.001	8.48 ± 1.48	7.78 ± 1.43	.065

Values are presented as mean ± standard deviation or n (%).

BBPS =Boston Bowel Preparation Scale, OST = oral sulfate tablet, PEG = polyethylene glycol.

* Visual analog scale score (0–10 points, 0: very bad, 10: excellent).

## 4. Discussion

In this prospective, randomized controlled study, we evaluated the efficacy and safety of a new tablet oral sulfate solution for bowel preparation before colonoscopy according to age. Our results showed that the use of OST is effective and safe for bowel preparation before colonoscopy, and it is useful to improve the visual field by reducing the bubbles in bowel lumen. In addition, we found that the use of OST is also effective and safe for bowel preparation before colonoscopy for elderly patients 65 years of age and older.

Adequate bowel preparation for efficient colonoscopy is not easy in clinical practice, because most preparation regimens can cause patient discomfort including nausea, vomiting, or abdominal distension/pain.^[19]^ A recently developed tablet formed oral sulfate solution, OST, has been expected to reduce patient discomfort and improve the quality of bowel preparation for colonoscopy. A prospective randomized controlled trial demonstrated that OST had similar efficacy, better tolerability, and lower incidences of nausea and vomiting compared with oral sulfate solution.^[16]^ In this study, we found that the OST group showed better bowel cleansing and bubble removal effects than the PEG group. Because the tolerability including satisfaction, difficulty for eating, and taste reported by the OST group was better than the PEG group, we presume that the bowel cleansing and bubble removal effects of OST were better than those of the PEG group. The simethicone included in the OST also seems to have contributed to better bubble removal effects.

With the development of medicine, most countries in the world have faced an increase in the aging population. In the Organisation for Economic Co-operation and Development data, the elderly population is defined as people 65 years of age and older.^[20]^ Because many elderly people have comorbidities, careful attention is needed for bowel preparation before colonoscopy for the elderly population. Until now, there have been no data on whether the OST is effective and safe for elderly 65 years of age and older. The strengths of our study are that this study included 61 elderly patients 65 years of age and older, 20 of whom were 70 years of age and older, and the oldest patient was 83 years. Our results showed that the bowel cleansing effect of the OST group was noninferior to the PEG group in elderly patients 65 years of age and older. In addition, we showed that the use of OST decreased more bubbles in the bowel lumen, and the tolerability including satisfaction, difficulty in eating, and taste reported by the OST group was better than the PEG group in elderly patients 65 years of age and older. Our data showed that the occurrence of adverse events including abdominal distension, abdominal pain, nausea, or vomiting was not different between the OST and PEG groups in elderly patients 65 years of age and older. These results suggest that the potential for adverse events of OST is similar to PEG. In this study, moderate and severe abdominal distension occurred in the OST group in patients under 65 years of age at a frequency of 11.7%, but these events were not reported in patients 65 years of age and older. Moderate and severe abdominal pain or nausea did not occur in the OST group of patients 65 years of age and older. Although the reason for these results is unclear, these findings suggest that the use of OST is relatively safe for elderly patients 65 years of age and older.

This study has some limitations. First, the number of patients 65 years of age and older was 61 (34.1%). We tried to include as many elderly patients as possible, but it was not easy because elderly patients tended to be reluctant to undergo colonoscopic examination. Further studies including more patients 65 years of age and older could strengthen our results. Second, only patients undergoing a colonoscopy for screening or postpolypectomy surveillance were included in this study. We could not evaluate the efficacy and safety of OST in patients with comorbidities, such as diabetes mellitus, hypertension, coronary artery disease, or renal failure. Therefore, these results may not be directly applicable to patients with comorbidities. Third, laboratory findings, including electrolytes, were not evaluated in this study. Therefore, we could not compare the change of electrolytes before and after bowel preparation between the 2 groups. Forth, patients taking medications for chronic constipation were not included in this study. Therefore, our results have no conclusion that can be drawn on the efficacy in patients with chronic constipation.

In conclusion, this randomized controlled study showed that the use of OST for bowel preparation before colonoscopy is as effective and safe as PEG, and these results were also observed in elderly patients 65 years of age and older. Although further studies will be needed to clarify these results, we expect that the OST could be a new good candidate for bowel preparation before colonoscopy regardless of age.

### Author contributions

Jae Hyun Kim: manuscript writing, manuscript reviewing and editing, conceptualization, visualization

Yong Eun Park: manuscript writing, conceptualization, investigation, data curation

Tae Oh Kim: manuscript reviewing and editing, investigation, methodology

Jongha Park: manuscript reviewing and editing, investigation, validation

Gyu Man Oh: data curation, investigation, methodology

Won Moon: manuscript reviewing and editing, data curation, visualization

Seun Ja Park: manuscript reviewing and editing, supervision, funding acquisition

All authors approved the final version of the manuscript and agreed to its submission.
